# Need for orthognathic surgery in cleft patients from Northern Finland

**DOI:** 10.2340/aos.v83.40338

**Published:** 2024-04-11

**Authors:** Mateusz Podleśny, Leena Ylikontiola, George K. Sándor, Ville Vuollo, Virpi Harila

**Affiliations:** a Research Unit of Population Health, Faculty of Medicine, University of Oulu, Finland; b Oral and Maxillofacial Surgeon, Oulu University Hospital, Finland. Medical Research Center Oulu, Oulu, Finland; c Oral and Maxillofacial Surgeon, Plastic Surgeon, Oulu University Hospital, Finland; d Medical Research Center Oulu, Oulu University Hospital, Oulu, Finland; e Orthodontist, Oulu University Hospital, Oulu, Finland

**Keywords:** Cleft lip, cleft palate, cleft lip and palate, orthognathic surgery, Le Fort, adolescent orthodontics

## Abstract

**Objective:**

Northern Finland has a unique distribution of clefts compared to the rest of Europe and Finland. This may reflect the need for orthognathic surgery among Northern Finland’s patient pool. The aim of this study was to compare previously operated patients aged 18 years or older with cleft lip, cleft lip and alveolus, cleft lip and palate, cleft palate and to evaluate the need for orthognathic surgery in order to achieve a stable and functional occlusion.

**Materials and methods:**

The study group consisted of all 18-years-old cleft patients treated in the Oulu Cleft Center. The total amount of patients was 110. The patients were compared retrospectively using patients’ hospital records. The majority of patients did not have any cleft-associated syndrome. The need for maxillary or bimaxillary orthognathic or corrective-jaw surgery was evaluated by the Oulu Cleft Team. A descriptive and statistical analysis was used to determine the need for orthognathic surgery according to sex and cleft type.

**Results:**

There were nineteen patients of the total of 110 patients who met the criteria requiring corrective-jaw surgery (17,3%). In total 12 males (25,0%) and 7 females (11,3%) were evaluated for the need of orthognathic surgery. Sixteen of the 19 patients had palatal involvement of the cleft.

**Conclusions:**

The need for orthognathic surgery was greater in the cleft lip palate and cleft palate patient groups compared to cleft lip alveolus or cleft lip groups. This study also found that males from Northern Finland need surgery more often than females.

## Introduction

Cleft lip (CL), cleft palate (CP), and cleft lip palate (CLP) are classified as facial malformations and oro-facial clefts with differing etiologies. The problems caused by the clefts affect the patient both physically and psychologically. Besides facial asymmetry, cleft patients may have problems with their dentition, occlusion, speech, and hearing.

In Finland, there is an exceptional distribution of cleft types compared to the rest of Europe and the Americas. There are also more CP patients in Northern Finland compared to other parts of Finland [1]. The total incidence of cleft patients in Finland is 2.56/1,000 children [1, 2]. Worldwide, approximately 1/1,000 children are born with a cleft; however, the number varies based on different ethnic groups and countries [3].

The cause of clefting is multifactorial, and the main cause remains unknown. In our study, the majority of patients had an isolated cleft palate (ICP) without any associated or known syndrome. The most studied etiologic factor is genetics. The risk of inheriting a cleft is approximately 4% in a family with a history of pre-existing cleft. The more clefts that the relatives have the higher the risk of clefting in the proband [4]. It is known that during the first trimester (weeks 5–12 and especially weeks 7 and 8 when the palate closes) are when possible clefts are formed. Environmental factors like passive and active smoking, exposure to different medications, alcohol consumption, pollution, vitamin deficiency, and late maternal age during pregnancy can affect the chances of clefting [5]. Chromosomal change in single genes are linked to syndromes [6]. Approximately 14.8%–59.5% of CP patients have some types of syndrome [7]. Those CP patients without any accompanying syndromal features are termed ICP. However, the majority of genes causing clefts are unknown despite clefts being a sign of a syndrome in over 300 syndromes [8]. The most common cleft-associated syndrome in Finland is Van der Woude syndrome, which occurs in approximately 2% of cleft patients [9].

The need for orthognathic surgery in cleft patients is determined by the original size of the cleft, the type, and severity of the malocclusion and its treatment options. It is common to wait for the skeletal growth to cease, before advancing to the orthognathic surgery phase.

The aim of this study was to determine how many over 18-year-old CL, CLA, CP, and CLP patients in this patient group had a good occlusion with only orthodontic treatment alone and how many needed or had orthognathic surgery done. The working hypothesis was that the larger the defect the more likely the patient will need future orthognathic intervention.

## Materials and methods

This study group consisted of all at least 18-year-old patients who were treated at Oulu Cleft Center (OCC). The total amount of the patients was 110. The data was collected retrospectively using patient’s hospital records. The detailed distribution of all the clefts in this study can be seen in Table 1. In the patient group, there was no history of alcohol or drug abuse during pregnancy. The study has been made according to the Helsinki declaration and approval was obtained from the Northern Ostrobothnia Hospital District (113/2021). The study group represents all of the clefts in Northern Finland. The patient inclusion criteria comprised the following: the patient had to be managed in OCC by the cleft team since the primary closure of the patient’s cleft and had reached the cessation of skeletal growth with the attainment of maturity. Patients who had moved abroad or to different hospital districts or moved to the OCC district after having started treatment in another hospital were excluded as well as the patients who did not meet the age criteria. In total, 16 patients were excluded from this study.

**Table 1:** Prevalence of different cleft types and sex in the study group.

Cleft type	M (%)	F (%)	Total (%)
CL	1 (0.9)	3 (2.7)	4 (3.6)
CLA	4 (3.6)	2 (1.8)	6 (5.5)
CP	24 (21.8)	39 (35.5)	63 (57.3)
CLP	11 (10)	10 (9.1)	21 (19.1)
SC	7 (6.4)	8 (7.3)	15 (13.6)
SC+CLA	1 (0.9)	0	1 (0.9)
Total	48 (43.6)	62 (56.4)	110 (100)

M: male; F: female; CL: cleft lip; CLA: cleft lip and alveolus; CP: cleft palate; CLP: cleft lip and cleft palate; SC: submucous cleft; SC + CLA: submucous cleft and cleft lip and alveolus.

All of the patients were managed by the same clinicians and treated by the same surgical team. Techniques used did not differ. Primary lip closure was done routinely at the age of 3 months and palatal closure took place between 9 and 12 months and was done using one-stage surgery. In total, 36 patients had speech surgery, and three speech surgeries had been redone again using another type of speech operation. Their regular dental care was received in primary health-care centers.

The need for maxillary or bimaxillary orthognathic surgery was later evaluated by the OCC. The criteria for surgery were related to occlusion: anterior crossbite, skeletal malocclusion, skeletal open bite, mandibular prognathism, and situations where conventional orthodontics would not be enough to correct deformities or malocclusion. Esthetics was not considered as the main criteria for jaw-correcting surgery. Patients with a syndrome were not evaluated for need of orthognathic surgery. Due to poor co-operation, patients with inadequate oral hygiene were not candidates for orthodontic treatment and therefore also not for orthognathic surgery. IBM SPSS Statistic software version 26.0 (IBM Corporation, Armonk, New York) was used for statistical analysis. A chi-square test was performed comparing proportions of various cleft types within sex and the need for orthognathic surgery. Threshold for statistical significance was set to 0.05.

The majority of patients had an isolated cleft without any relation to syndromes. All patients were first treated orthodontically during childhood and adolescence. Quad Helix was used by 47 patients, trans-palatal arch by 28 patients, 80 patients had fixed appliances, and seven had facemasks. In addition, 16 children had activators and 17 used headgear.

## Results

A total of 19 (17.3%) patients required orthognathic surgery; however, eight patients declined surgical treatment. From the 110 patients, only four patients (3.6%) had undergone Le Fort 1-surgery (one female/three males), two patients (1.8%) had a surgically assisted rapid maxillary expansion (SARME) procedure (one female and one male). There were no two-jaw bimaxillary surgeries in this patient group. Five patients (4.6%) were planned to have a Le Fort 1-osteotomy within a year although the patients did not have their surgeries as of yet (Table 2).

**Table 2:** The need for surgery for cleft patients.

Need for surgery (%)	DO (%)	SARME (%)	Bimax (%)	LeFort 1 (%)	Planned surgery (%)
19 (17.3)	1 (0.9)	2 (1.8)	0	4 (3.6)	5 (4.6)

DO: distraction osteogenesis; SARME: surgically assisted rapid maxillary expansion; Bimax: bimaxillary surgery.

The need for surgery was analyzed according to cleft type and sex (Table 3). This infers that patients with a specific cleft type need surgical correction of jaw relation more often than those with lesser defects (*p* < 0.001). However, the patients who were found to need orthognathic surgery all had received presurgical orthodontic treatment with fixed appliances, Quad Helix but only two had a history of facemask usage. In addition, males had a greater need for surgery than females (*p* = 0.059). Table 4 shows the detailed need for surgery by cleft types in corresponding patient groups. There was no statistical difference between the distribution of different cleft types and sex (*p* = 0.461).

**Table 3:** The need for surgery by cleft type and sex.

Cleft type	M (%)	F (%)	Total (%)
CLA	2 (10.5)	0	2 (10.5)
CP	3 (15.8)	2 (10.5)	5 (26.3)
CLP	6 (31.6)	5 (26.3)	11 (57.9)
SC	1 (5.3)	0	1 (5.3)
Total	12 (63.2)	7 (36.8)	19 (100.00)

M: male; F: female; CL: cleft lip; CLA: cleft lip and alveolus; CP: cleft palate; CLP: cleft lip and cleft palate; SC: submucous cleft; SC + CLA: submucous cleft and cleft lip and alveolus.

**Table 4:** The need for surgery by cleft types in corresponding patient groups.

Cleft type	*n*	M (%)	F (%)	Planned (%)
CL	4	0	0	0
CLA	6	2 (33.3)	0	2 (33.3)
CP	63	3 (4.8)	2 (3.2)	5 (7.9)
CLP	21	6 (28.6)	5 (23.9)	11 (52.4)
SC	15	1 (6.7)	0	1 (6.7)
SC+CLA	1	0	0	0
Total	110	12 (25.0)	7 (11.3)	19 (17.3)

n: number of specific cleft types in the study; x: total incidence of planned surgeries; M: male; F: female; CL: cleft lip; CLA: cleft lip and alveolus; CP: cleft palate; CLP: cleft lip and cleft palate; SC: submucous cleft; SC + CLA: submucous cleft and cleft lip and alveolus.

## Discussion

A major finding of this study is that male cleft patients need surgery more often than females. This might be due to differences in mandibular growth and its timing. Also seven patients out of 110 had facemask as part of orthodontic treatment and only two of those facemask patients needed orthognathic surgery in adulthood. This suggests that facemask may have a preventive effect due to promoting maxillary growth and therefore diminishing the difference between maxilla and mandible.

The advancements in early orthodontics may have a significant role in diminishing the need for orthognathic surgery. Another finding was that these results differ slightly from previously reported studies. Generally, CLP patients are more in need of orthognathic surgery than CL or CP patients [10]. It seems that Northern Finland’s CLP and also CLA patients need surgery more often than other cleft types. Isolated CP patients are more likely to be treated without the need for orthognathic surgery. In a study by Gustafsson et al. conducted on patients from Southern Finland, a total of 33.3% of CLP patients required orthognathic surgery at a median age of 17.6 years [11]. Further studies are needed to show whether alveolar scar tissue in CLA patients diminishes maxillary growth compared to non-alveolar lip clefts. The differences may be caused by a variety of inherited ethno-genetic factors and differing natural jaw proportions found in different ethnic groups. Seven patients did not want to have orthognathic surgery. The major reasons for patients to decline orthognathic surgery were self-acceptance, completion of compulsory military service, asymptomatic status, and lack of interest in additional treatment as the patients have had intensive orthodontic treatment during adolescence.

Early primary palate surgery does not seem to affect the need for future orthognathic surgery, but patients treated with primary lip closure in CLP have a tendency to require future orthognathic treatment. Park et al. concluded that later lip closure can allow the avoidance of orthognathic surgery[12]. In the study by Ore et al. in 2017, 16.9% of CL and/or palate patients underwent orthognathic surgery. Approximately, one third of CLP patients need orthognathic surgery at the end of growth. Even though orthodontic treatment is important for function, aesthetics, and rehabilitation, long orthodontic treatment may not affect craniofacial growth in the long term in CLP patients [13]. New studies report that improved treatment techniques have diminished the need for orthognathic surgery from 60% to 47% in cleft patients [14]. A recent Korean study reports that orthognathic surgery is more likely with increasing cleft severity. In that study 8.5% of CL and alveolar clefts, 21.4% of unilateral CLP, and 30.0% of bilateral CLP patients needed some type of orthognathic surgery [15]. In the future, Holdaway angle and Witts appraisal together could be used as markers for determining the need for orthognathic surgery in Angle class III patients [16].

Orthognathic treatment has a large improvement in the quality of life [17]. In addition, the aim is to evenly place teeth to achieve normal bite relationships and cranial proportions [18]. Surgery may include maxilla, mandible, or both. Nowadays orthognathic surgery is safe, and the results are quite predictable [19]. Le Fort I -surgeries are safe and stable treatment options [20]. However, maxillary advancement in cleft patients has its drawbacks. It is possible for the speech to develop postoperative hypernasality [21]. This happens due to the soft palate moving forward following the bony advancement of the maxilla and is difficult to predict [22]. Segmental Le Fort I-osteotomies can balance the occlusion and skeletal relations, though it is studied mainly in patients without clefts. In a study by Watts et al., both segmental and conventional Le Fort I-surgeries had a similar relapse, and there were no significant differences in the healing process [23].

SARME is a procedure where the maxilla is transversally expanded after the end of growth. SARME is considered safe and stable [24], and the results are well predicted. Also, the long-term skeletal and dental effects are good [25]. According to the literature review, the largest expansion point is at the molar site [26]. Also, expansion leads to enlargement of nasal floor width resulting in increased transversal proportions and most likely improvement in breathing function [27].

The majority of cleft patients in this study occurred without an associated syndrome, and their clefts were classified as isolated or non-syndromic. This lack of syndrome association might be due to Northern Finland’s uniquely exceptional patient and gene pool. On the other hand, this study is limited by a quite small population group.

## Conclusion

This study suggests that Northern Finland’s male cleft patients need orthognathic surgery over twice as often as females. The more severe the defect the more likely adulthood orthognathic surgery is needed. Isolated CP has a significantly lesser effect on dentition compared to other clefts. Early stimulation for maxillary growth such as facemasks may have a significant role in reducing the need for surgery. However, further studies are needed on larger populations as this study is limited by a small study group.
